# Understanding the Mechanism of Translocation of Adenylate Cyclase Toxin across Biological Membranes

**DOI:** 10.3390/toxins9100295

**Published:** 2017-09-21

**Authors:** Helena Ostolaza, César Martín, David González-Bullón, Kepa B. Uribe, Asier Etxaniz

**Affiliations:** Biofisika Insititute (UPV/EHU, CSIC) and Department of Biochemistry and Molecular Biology, University of Basque Country (UPV/EHU), 48080 Bilbao, Spain; cesar.martin@ehu.eus (C.M.); david_go88@hotmail.com (D.G.-B); kepa1985@gmail.com (K.B.U); aetxaniz7@gmail.com (A.E.)

**Keywords:** Adenylate cyclase, RTX toxin, protein translocation, phospholipase activity, model membranes

## Abstract

Adenylate cyclase toxin (ACT) is one of the principal virulence factors secreted by the whooping cough causative bacterium *Bordetella pertussis*, and it has a critical role in colonization of the respiratory tract and establishment of the disease. ACT targets phagocytes via binding to the CD11b/CD18 integrin and delivers its N-terminal adenylate cyclase (AC) domain directly to the cell cytosol, where it catalyzes unregulated conversion of cytosolic ATP into cAMP upon activation by binding to cellular calmodulin. High cAMP levels disrupt bactericidal functions of the immune cells, ultimately leading to cell death. In spite of its relevance in the ACT biology, the mechanism by which its ≈400 amino acid-long AC domain is transported through the target plasma membrane, and is released into the target cytosol, remains enigmatic. This article is devoted to refresh our knowledge on the mechanism of AC translocation across biological membranes. Two models, the so-called “two-step model” and the recently-proposed “toroidal pore model”, will be considered.

## 1. Introduction

**Figure 1:**
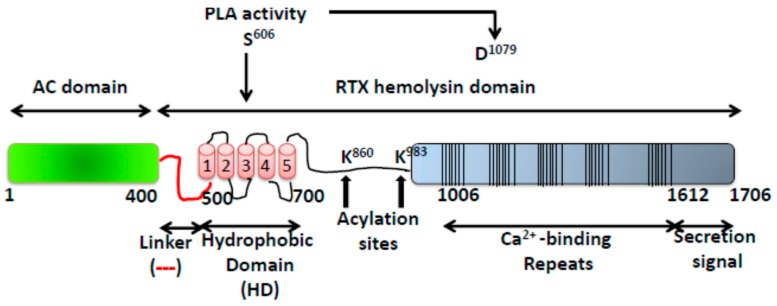
Structural organization of the ACT toxin. The catalytic domain (AC domain) (in green) extends approximately from residues 1 to 400. The RTX haemolysin domain (residues from ≈500 to 1706) contains the hydrophobic domain (HD) constituted by five hydrophobic-amphipathic alpha-helices (in red), two conserved acylation sites, K860 and K983, required for activation by palmitoylation mediated by CyaC acyltransferase, and five blocks formed by low affinity calcium-binding repeats. The Ca^2+^-binding region (residues 1006–1612) is denoted by multiple lines, with each line corresponding to single nonapeptide repeats with consensus sequence GGXGXDXLX. The segment between residues 1638–1706) corresponds to the C-terminal secretion signal.

The whooping cough bacterium *Bordetella pertussis* secretes several virulence factors, and among them the Adenylate Cyclase Toxin (ACT or CyaA) is crucial in the early steps of colonization of the respiratory tract by the bacterium [1] ACT belongs to an extensive family of toxins, referred to as RTX (Repeat in ToXin), that are synthesized by Gram-negative organisms sharing several functional and genetic features [2]. Synthesis, maturation and secretion of ACT are determined by the *CyaCABD* operon [3]. Gene product A is a 1706 amino acid polypeptide corresponding to a protoxin (pro-ACT) that matures in the bacterial cytosol to the active form (ACT) by CyaC-directed acylation at two conserved internal lysine residues (Lys 863 and Lys 983) [4,5]. ACT is then secreted across both bacterial membranes by the type I secretion system (T1SS) employing an uncleaved C-terminal recognition signal and a specific secretion machinery [3]. Like other members of the RTX family, ACT is extracellularly secreted as a soluble protein [6], but also has the ability to interact and insert into biological membranes. Aside these similarities, ACT is distinguished from the rest of RTX toxins by bearing a cell-invasive N-terminal enzymatic adenylate cyclase (AC) domain (~400 residues) fused to a C-terminal haemolysin (Hly) moiety (residues ≈400–1706) characteristic of the RTX family (Figure 1) [3,6]. The haemolysin domain contains in turn: a hydrophobic region (HD) with five predicted hydrophobic/amphipathic α-helices (~500–700 residues) likely involved in pore-formation; two conserved Lys residues (Lys 863 and Lys 983) that are post-translationally fatty acylated [4,5]; a series of calcium-binding repeats (~40) that are formed by Gly- and Asp-rich nonapeptides harbouring a conserved G-G-X-G-X-D-X-(L/I/V)-X motif [7,8,9] and finally, a C-terminal secretion signal recognized by the secretion proteins (CyaB, CyaD, and CyaE proteins) [3,4] (Figure 1). All known ACT activities require binding of Ca^2+^ ions into the mentioned calcium-binding sites whose affinity constant for the cation is in the mM range [8,9]. The repeats domain has been reported to also be involved in the toxin binding to its specific cellular receptor, the CD11b/CD18 integrin (*α*M*β*2, Mac-1, or CR3) expressed on myeloid phagocytes [10,11].

**Figure 2:**
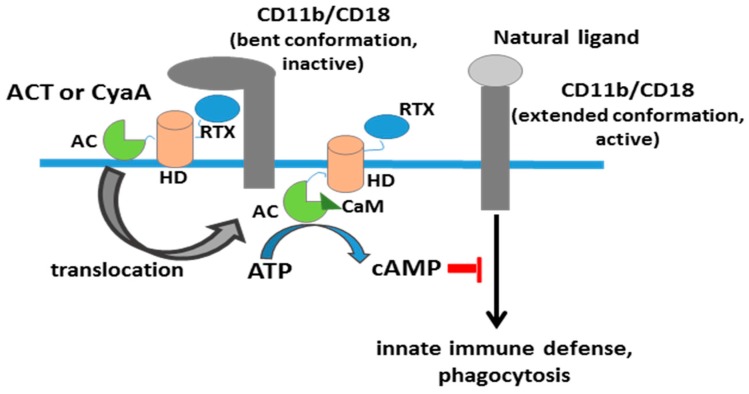
Scheme of adenylate cylase toxin activity. Simplified schematic depiction of the stages of adenylate cyclase toxin interaction, insertion and translocation of the AC domain through the plasma membrane of monocytic cells expressing the toxin receptor CD11b/CD18 (*α*M*β*2, Mac-1, or CR3). ACT is believed to bind presumably to the inactive (bent) conformation of the integrin CR3 [18], while natural physiological ligands (fibrinogen, iC3b, intercellular adhesion molecule 1(ICAM-1), and other protein ligands) bind to the active (extended) conformation of CR3 [19]. AC, adenylate cyclase domain, in light green; HD, hydrophobic domain, in red colour; RTX, calcium-binding repeats domain, in blue; CaM, calmodulin, in dark green.

Upon binding to its receptor, ACT invades these immune cells by delivering directly into their cytosol a calmodulin-activated adenylate cyclase (AC) domain. This AC domain catalyses the uncontrolled conversion of cytosolic ATP into cAMP [12], which causes inhibition of the oxidative burst and complement-mediated opsonophagocytic killing of bacteria (Figure 2) [13,14]. ACT has also haemolytic properties, conferred by its C-terminal domain, which may form pores that permeabilize cell membrane for cations, contributing to overall cytoxicity of ACT towards phagocytes [15,16,17].

*Bordetella* adenylate cyclase is, together with anthrax toxin edema factor (EF), the prototype of a bacterial protein toxin that attacks the immune response by overboosting the major signalling pathways in the immune response, the cAMP and MAPK pathways, upon activation of its adenylyl cyclase activity by calmodulin [20,21,22,23]. However, the mechanism by which these two toxins enter to the target cell cytosol to produce cAMP levels seems to be very different [20]: anthrax toxin follows a receptor-mediated endocytosis, followed by the transport of the oedema factor from the endosome to the cytosol, through a specific heptameric protein channel formed by the protective antigen (PA) subunit [22], whereas ACT internalizes its catalytic AC domain directly, from the hydrophilic extracellular medium into the hydrophobic environment of the plasma membrane lipid bilayer, and then, to the cell cytoplasm [6,21,24,25,26]. How this is accomplished at the molecular level remains enigmatic. In this review we will briefly refresh the previous knowledge about AC translocation and the models postulated, and include the most recent findings.

## 2. Previous Ideas about AC Translocation and Models Postulated

Almost thirty years have passed since Gordon and colleagues [27] suggested that the AC domain translocation across the lipid bilayer of cellular membrane is not preceded by toxin endocytosis, which is consistent with the rapid kinetics (a few tens of seconds) detected for the intracellular cAMP accumulation in the ACT-treated cells [24]. A few years later, the first translocation model (“pore model”) was posited [24] in which the COOH-terminal portion of the toxin (RTX haemolysin domain) would create in the membrane a channel through which the NH_2_-terminal fragment (AC domain) would be translocated. However, this model was rapidly discarded after the publication of a biophysical study of conductance measurements in planar lipid bilayers [17] in which it was claimed that the pore formed by the ACT-haemolysin moiety was apparently too narrow (0.6–0.8 nm diameter) as to allow the passage of the ≈40 kDa N-terminal AC domain polypeptide [17]. Other studies of haemolysis with different osmotic protectants seemed consistent with the idea that the ACT pore was very small [15] and, together, form part of the “non-pore” tendency in the field to explain AC translocation. Linked to this is the idea prevailing in the field that AC translocation does not depend on membrane permeabilization by ACT pores, or in other words, that AC translocation and pore formation by ACT toxin are independent processes [28]. This conclusion has been mainly based on mutational studies in which it was shown that mutants in the ACT pore-forming domain (E570Q) or the acylation domain (K860R) selectively ablate the pore-forming activity of ACT while, at the same time, the capacity of such ACT mutants to translocate the AC domain across the cytoplasmic membrane into the cytosol of macrophage cells and to elevate cellular cAMP concentrations remained intact [28]. It is in this same sense that the results with other ACT mutants in specific residues within two of the five amphiphatic α-helices, which are supposed to form part of the toxin hydrophobic pore-forming domain (E509 and E516 in helix 1, E570 and E581 in helix 3), have been shown to affect both translocation and pore formation [26,29]. Charge reversal by Lys substitutions of the E509 or of the adjacent E516 residue reduces the capacity of the toxin to translocate the AC domain across membrane and enhances significantly its specific haemolytic activity and channel-forming capacity in lipid bilayer membranes [29]. Combination of the E509K and E516K mutations in a single molecule further exacerbates haemolytic and channel forming activity and ablates translocation of the AC domain into cells. Replacement of E516 by positively-charged lysine residue (E516K) increases the propensity of ACT to form pores, whereas proline (E516P) or glutamine (E516Q) substitutions extend the lifetime of open single pore units. All three substitutions also cause a drop of pore selectivity for cations [29]. Substitutions of E570 and E581 by helix-breaking proline or positively-charged lysine residue reduce (E570K, E581P) or ablate (E570P, E581K) AC membrane translocation. Moreover, E570P, E570K, and E581P substitutions down-modulate also the specific haemolytic activity of ACT. In contrast, the E581K substitution enhances the haemolytic activity of ACT four times, increasing both the frequency of formation and lifetime of toxin pores. These data have led to the idea that in spite of their independency, several structural elements might be shared by both processes, translocation and pore-formation, and also to the proposal that two distinct ACT conformers, forming in parallel and existing in equilibrium, insert into target cell membrane [28]. One would be the translocation precursor that would account for delivery of the AC domain across the lipid bilayer and provokes also a concomitant influx of calcium ions into cells. The other conformer would form a pore precursor that would oligomerize into ACT pores, provoking potassium efflux from target cells [28]. Other characteristics of AC translocation have been reported such as its strict calcium dependence at mM range [30,31,32,33], temperature above 15 °C [27], negative membrane potential [33,34], monomeric stoichiometry [35], or unfolding of the AC domain. An intriguing feature of AC translocation is the extraordinary versatility of the AC domain to transport into the cytosol of CD11b^+^ target cells large heterologous cargo polypeptides of up to 200 residues in length within the AC domain [36,37,38].

### Two-Step Model for Membrane Penetration

Ten years ago it was reported that ACT induced extracellular Ca^2+^ entry into target cells independently of the ACT pore, and that the translocating AC polypeptide itself would participate in formation of a novel type of membrane path for calcium ions [39]. Three years later the same laboratory published other work proposing a calcium-dependent two-step model for AC translocation into phagocytes [40]: in a first step, and upon ACT binding to its CD11b/CD18 integrin receptor through its repeats domain, the hydrophobic segments of ACT could insert into the plasma membrane to anchor the toxin to the target cell. Then a membrane “translocation intermediate” of the AC polypeptide would itself participate in formation of a novel type of a “transiently opened path” across the cytoplasmic membrane that would conduct extracellular calcium ions into the cytosol of monocytic cells [39,40]. This calcium influx results in a calpain-mediated cleavage of talin, which anchors the CD11b/CD18 integrin to the cytoskeleton. The ACT-integrin complex would then be free to diffuse and relocate into cholesterol-rich lipid microdomains, where the sterol-rich environment could trigger the final step of the AC domain translocation across the cell membrane [40]. Thus, the calcium-dependence derives from a cellular event required for the relocation of membrane-bound ACT toxin rather than a direct effect on AC activity. Recently, we have observed in the laboratory that, at low toxin doses (≤100 ng/mL) the ACT-induced Ca^2+^ influx into macrophages is practically null (Etxaniz et al., 2017; manuscript in revision). This is thus a weak point of the two-step model, since it cannot explain in a satisfactory way how the toxin-receptor complex can relocate into lipid rafts at low ACT concentrations, at which cell intoxication by cAMP is particularly relevant for cell intoxication by ACT.

Other issues await a satisfactory response, such as how can ACT translocate its AC domain into cells that do not express the CD11b/CD18 integrin receptor [41,42,43] or how can ACT successfully transport, upon fusion to the C-terminal ACT-hemolysin domain, such variety of N-terminal polypeptides carrying so different sequences and lengths [36,37,38]? A path that has to indistinctly assist the translocation of a 400 amino acid-long chain, or of other different polypeptides, has to be expectedly dynamic, adaptable, and versatile. Recent work in which a novel ACT activity has been discovered, namely, an intrinsic phospholipase A activity with membrane remodelling capability [44], may provide, as we shall consider in the next sections, an answer to these questions.

## 3. Interaction of the AC Domain with Membranes and Role of the Region Adjacent to the Catalytic Domain in AC Translocation

The ACT segment bearing adenylate cyclase activity (AC domain) extends approximately between residues 1 and 390 of the toxin polypeptide chain. The crystal structure of this AC domain in complex with the C-terminal domain of calmodulin was published more than a decade ago [45]. The isolated AC domain in solution is globular with a rather high hydration and its secondary structure is an α/β mixture [46]. About one third of the amino acids in its sequence are charged, negatively (E + D = 59) or positively (K + R = 53).

A fundamental premise for any polypeptide segment that has to cross directly the cell membrane to get into the cytosol is that it has to be transiently inserted into the lipid bilayer. The isolated AC domain does not, however, appear to have this ability [47]. A protein covering the entire catalytic domain, AC384 (encompassing residues 1–384 of ACT) does not exhibit membrane association capability [47]. However, a longer polypeptide, encompassing residues 1 to 489 (AC489) of ACT has been recently shown that it is able to interact with lipid membranes and induce lipid bilayer destabilization [47], and that deletion of residues 375 to 485 within ACT completely abolishes the intoxication process of eukaryotic cells, leading to the conclusion that this segment between the catalytic and the hydrophobic domains has a critical role in AC translocation [47]. Previously, it had been noted that translocation of the catalytic domain could be blocked by a monoclonal antibody that recognizes an epitope located between residues 373 and 399 of the ACT polypeptide [48], which seems consistent with the mentioned hypothesis.

**Figure 3:**
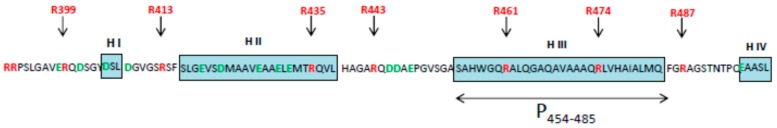
Scheme of the linker segment. Schematic representation of the amino acid sequence corresponding to the linker segment (aa 390–500) of the sequence of the ACT toxin (Acc ni 6092563 emb CAB59146.1, which includes a prediction of the most probable secondary structure elements that was determined with the I-Tasser predictor [50]. The peptide segment extending between residues 454 and 485 (P_454–485_) has been indicated with a double-head arrow. In red appear mutated arginines, and in green, mutated aspartic and glutamic acids [51].

The segment that joins the AC domain with the hydrophobic domain of ACT, which we will refer to as “linker segment” has attracted the attention of several researchers in the last years. The ACT linker segment has no homologs in the other toxins of the RTX family. In Figure 3 it is shown a schematic representation of the amino acid sequence of this linker segment with predicted secondary structure elements (α-helices H I to H IV in blue), so that the data detailed below can be more easily understood. Within the linker region it has been identified a peptide segment (residues 454 to 485, in helix H III) (Figure 3) with membrane-active properties [49], and a synthetic peptide corresponding to this sequence (P454, residues 454–485, in helix H III) has been characterized by various biophysical approaches, finding that it binds to membranes containing anionic lipids, adopts an α-helical structure oriented in the plane with respect to the lipid bilayer and permeabilizes lipid vesicles [49]. These results have led to propose that the region encompassing the helix 454–485 of ACT may insert into target cell membrane and induce a local destabilization of the lipid bilayer, favouring the translocation of the catalytic domain across the plasma membrane [49].

Interestingly, it has been recently found that the linker segment appears not only to be involved in AC translocation, but it appears to modulate the pore-forming activity of ACT [51]. It was observed that individual substitutions of the positively-charged residues within the linker segment (arginines 435, 443, 461, 474, and 487) (see Figure 3) by neutral alanines reduce by about 20% to 40% the specific capacity of these mutant toxins to translocate their AC domains across the erythrocyte membrane while exhibiting the full capacity to bind and permeabilize erythrocytes (haemolytic activity) [51]. Substitutions of the negatively charged residues E419, D422, E427, E430, E432 (in helix II, residues in green), and D445, D446, and E448 (between helix II and III, residues in green), instead, strongly enhance the specific haemolytic activity of the respective toxins on erythrocytes, and affect the conductance properties of single ACT pores in black lipid membranes, whereas their cell-invasiveness remains intact [51]. Much earlier, it had been noted the involvement of the linker segment in modulation of ACT pore formation, given that a truncated construct lacking the N-terminal residues 6–489 (ΔN489) exhibited a strongly enhanced pore-forming and cell-permeabilizing activity [48]. All these data together with previous results demonstrating that translocation of the AC domain requires a negative membrane potential [33,34] and a net positive charge in the AC domain [52] have recently led to the hypothesis that the translocated polypeptide may be conducted along a “negatively-charged hydrophilic surface” across the membrane lipid bilayer, which would possibly comprise the amphipathic α-helices formed by residues 502 to 522 (helix 1) and 565 to 591 (helix 3) of the toxin hydrophobic domain (HD) (see Figure 1) [51]. Previous findings that helix-breaking or charge-reversing substitutions in these helices (E509K, E516K, and E581K) drastically reduce AC translocation, while potentiating the pore-forming capacity of ACT might support such hypothesis [28,29]. It has also been postulated that clusters of negatively-charged residues in the N-terminal half of the linker segment would not form part of the conducting path along which the AC domain translocates across the lipid bilayer, as the reduction of the net negative charge of that N-terminal half from −7 to −1 (e.g., in ACT mutant D445K + D446K + E448K), or the reversal of its overall charge to +3 in the ACT mutant E419K-E432K construct, have essentially no impact on the capacity of ACT to translocate its AC domain across erythrocyte or macrophage membranes [51]. What it is not so easy to envisage is how a “negatively-charged hydrophilic surface” formed by helices 1 and 3 of the ACT hydrophobic domain, as it is proposed, can be favourably exposed in the interior of the lipid bilayer surrounded by the hydrocarbon chains of phospholipid fatty acids unless it forms part of the lumen of some kind of pore. In the next section, we shall see an alternative mechanism for AC domain translocation in which a novel intrinsic phospholipase A activity of ACT has a prominent role.

## 4. Intrinsic Phospholipase A Activity of ACT and Its Role in AC Domain Translocation

Recently it has been revealed that ACT is a calcium-dependent phospholipase A [44], sharing sequence similarity with members of the patatin-like phospholipases and mammalian cPLA_2_ [53]. Two conserved active-site catalytic motifs, namely, GAS^606^AG sequence (consensus motif GXSXG) and D^1079^GG sequence (consensus motif DXG/A) have been identified in the toxin sequence and it has been proved that Ser-606 and Asp-1079 are essential catalytic residues in the ACT-PLA_1_ activity, validating that ACT is a serine acylhydrolase [44]. ACT can hydrolyse both the *sn-1* and the *sn-2* ester bonds of glycerophospholipid substrates, releasing into the membrane free fatty acids and lysophospholipids. However, it has no lysophospholipase activity; the toxin cleaves with notably greater efficiency the *sn-1* ester bond of glycerophospholipid substrates than the *sn-2* one, suggesting that ACT most likely acts as a PLA_1_ on natural membranes [44]. ACT hydrolyses in vitro both neutral and anionic phospholipipds, with similar efficiency, suggesting that ACT-PLA may not have a clear substrate specificity for none given phospholipid, in the likeness of the patatin PLA enzyme [54]. ACT also releases free fatty acids from the macrophage cell membrane, in the same toxin concentration range that it results in cytotoxicity for cells [44].

A significant contribution of the mentioned study has been the elucidation of the biological consequence of this novel ACT-PLA activity. It has been demonstrated that ACT-PLA activity is directly linked to the AC translocation across the plasma membrane of target phagocytes [44]. Consistently with this essential role of the ACT-PLA activity in AC transport it has been found that ACT-induced cell toxicity dramatically decreases in the macrophages treated with PLA-deficient ACT mutant proteins, particularly at the toxin concentration-range close to biological reality of bacterial infection (<100 ng/mL), indicating that PLA activity is instrumental for ACT cytotoxicity [44].

### Model of Toroidal Pore

**Figure 4:**
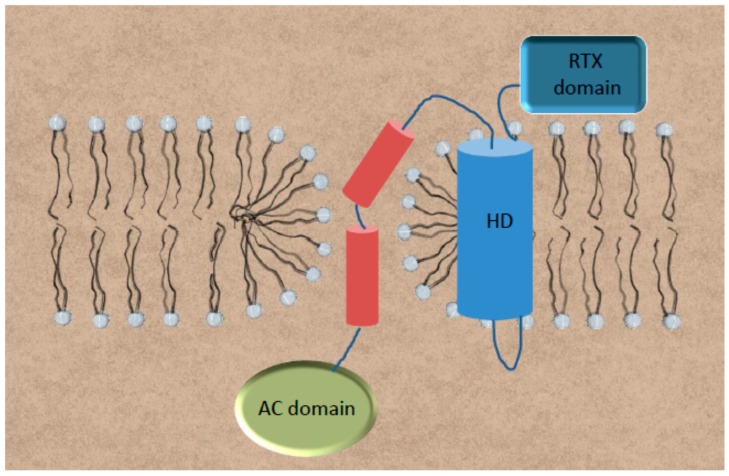
Schematic depiction of the PLA-based toroidal model for AC translocation. We hypothesize that in situ generation of non-lamellar lysophospholipids by the ACT-PLA activity on plasma membrane phospholipids would form a hydrophilic lipid pore of the toroidal architecture through which the AC domain transfer could directly take place. Such a hydrophilic lipidic pore might be of proteolipidic nature, this is, with the pore walls likely constituted by both lipids and one, or perhaps more, helices of the pore-forming region of ACT. The different structural elements are not drawn to scale. Colours used: In light blue, the whole hydrophobic domain which has been represented as a single big helical element; in dark blue, the RTX domain; in red, the linker segment, in which the two longer helices have been depicted; and in green, the AC domain, once in the cytosol.

The reaction products of ACT-PLA activity on plasma membrane phospholipids are lysophospholipids and free fatty acids [55]. Lysophospholipids, with its positive spontaneous curvature, have been proved to induce in membranes formation of hydrophilic permeable proteolipidic “toroidal pores” in which amphipathic peptides and proteins are inserted. This is due to a decrease in line tension within the lipid bilayer because of relief of curvature stress along the membrane edge of the pore wall [56,57,58,59]. A toroidal pore is characterized by the fusion of the outer and inner bilayer leaflets to form a continuous surface at the edge of the pore, which is formed by both lipids and proteins. In a toroidal pore therefore its constituent monolayers become continuous via the pore-lining lipids, allowing the movement of lipid molecules from one monolayer of the bilayer to the other [56,57,58,59]. ACT-PLA activity has been proved to be directly involved in promoting transbilayer lipid motion both in pure phosphatidylcholine liposomes and also in macrophages [44,60], indicating that ACT-PLA activity could indeed form toroidal pores in the lipid bilayer. All these data have led to propose a different model for AC translocation, in which the in situ generation of non-lamellar lysophospholipids by the ACT-PLA activity on plasma membrane phospholipids would form a hydrophilic lipid pore of toroidal architecture through which the AC domain transfer could directly take place (Figure 4). It is plausible to envisage that such a hydrophilic lipidic pore might be of a proteolipidic nature, constituted likely by both lipids and one or more helices of the pore-forming region; indeed, location of the ACT-PLA catalytic Ser-606 within helix4 (see Figure 1), one of the predicted α-helices of the so-called hydrophobic pore-forming domain of ACT, seems consistent with this idea. The previous findings mentioned above that mutations in specific amino acids of this and other α-helices from the hydrophobic domain impair not only the haemolytic activity, but also the translocation capacity of ACT [29] agree also with this proposal. This toroidal model for AC translocation constitutes also a plausible explanation for the extraordinary versatility of the AC domain to transport into the cytosol of CD11b^+^ target cells large heterologous cargo polypeptides of up to 200 residues in length within the AC domain [36,37] since, among the unique characteristics of the proteolipidic toroidal pores, are the structural flexibility, low selectivity, and variable size [58,59].

Direct participation in AC domain translocation of an ACT-PLA-generated hydrophilic toroidal tunnel, in which the pore-walls may in part be lined by phospholipid head groups, may also be consistent with previous observations showing a net positive charge requirement for AC domain to be translocated [52]. Moreover, the possible formation of ion couples among those positively-charged amino acid lateral chains with negatively charged phospholipid and/or fatty acid headgroups exposed at the pore walls may represent an energy source that might contribute to lowering the energy barrier for the AC domain insertion within the lipid bilayer [61,62]. Very interestingly, a “product inhibition” or “feedback-like” mechanism has been found to operate in the ACT-PLA activity [44], which may be very important for the ACT-PLA mechanism, since it suggests that the ACT-PLA activity is not, to massively “destroy” the target cell outer membrane lipids, but, quite the opposite, that this ACT lipid hydrolytic activity has likely evolved as a “security mechanism” by inducing a local, subtle and “surgical” effect on the cell membrane of the target cells, necessary to transport the AC domain from the extracellular milieu to the cell interior. It has also been of great relevance the finding that ACT-PLA activity needs calcium in the millimolar (mM) range to be fully operative [44], providing a straightforward explanation of the strict calcium dependence of the AC translocation observed by numerous laboratories over time [30,31,42]. It might be speculated that the requirement of a co-factor, such as calcium ions, for the ACT-PLA activity might be linked to the absence of toxicity of ACT when expressed in bacteria, acting somehow as a “security mechanism” for the prokaryotic cell.

## 5. Conclusions

After more than thirty years we still do not know what the molecular details of the ACT catalytic domain delivery into the cell cytosol are and how the 400 amino acid-long AC domain crosses the cell membrane exactly. For a long time the determination of very low single-channel conductance values for the pore presumably formed by ACT in lipid bilayers has strongly conditioned the field, prevailing the idea of a direct “non-pore model” for AC translocation. The most recent discovery that ACT is, itself, a phospholipase A and that such lipid hydrolytic activity is critical for AC domain delivery, together with the postulation of a new “toroidal pore model” has opened a debate of pore-yes, pore-not, to explain the AC transport, offering exciting possibilities to investigate new questions that arise: which are the exact protein segments that conform the proteolipidic toroidal pore for AC transport? Is the toroidal pore involved only in AC transport, or is it also responsible of the ACT-induced permeabilization and haemolysis? Might the AC domain itself be capping the lumen of the ACT pore? Could a toroidal pore be also involved in cell membrane permeabilization by other homologous RTX toxins in the family? The new discovery of ACT-PLA activity opens a new window not only to continue investigating the mechanistic and molecular details of AC transport across cell membranes, but also to explore how this ACT-PLA activity can be regulated and, thus, opens new possibilities for the therapeutic control of whooping cough, an infectious disease that is the fifth largest cause of vaccine-preventable death in infants. We envision coming years of exciting research to achieve major progress in our understanding of PLA-based AC transport across biological membranes and how to modulate it.

## References

[1] Goodwin M.S.M., Weiss A.A. (1990). Adenylate Cyclase Toxin is Critical for Colonization and Pertussis Toxin is Critical for Lethal Infection by *Bordetella pertussis* in Infant Mice. Infect. Immun..

[2] Welch R.A. (1991). Pore-Forming Cytolysins of Gram-Negative Bacteria. Mol. Microbiol..

[3] Glaser P., Sakamoto H., Bellalou J., Ullmann A., Danchin A. (1988). Secretion of Cyclolysin, the Calmodulin-Sensitive Adenylate Cyclase-Haemolysin Bifunctional Protein of *Bordetella pertussis*. EMBO J..

[4] Hackett M., Guo L., Shabanowitz J., Hunt D.F., Hewlett E.L. (1994). Internal Lysine Palmitoylation in Adenylate Cyclase Toxin from *Bordetella pertussis*. Science.

[5] Barry E.M., Weiss A.A., Ehrmann I.E., Gray M.C., Hewlett E.L., Goodwin M.S.M. (1991). *Bordetella pertussis* Adenylate Cyclase Toxin and Hemolytic Activities Require a Second Gene, cyaC, for Activation. J. Bacteriol..

[6] Ladant D., Ullmann A. (1999). *Bordetella pertussis* Adenylate Cyclase: A Toxin with Multiple Talents. Trends Microbiol..

[7] Bauche C., Chenal A., Knapp O., Bodenreider C., Benz R., Chaffotte A., Ladant D. (2006). Structural and Functional Characterization of an Essential RTX Subdomain of *Bordetella pertussis* Adenylate Cyclase Toxin. J. Biol. Chem..

[8] Rose T., Sebo P., Bellalou J., Ladant D. (1995). Interaction of Calcium with *Bordetella pertussis* Adenylate Cyclase Toxin. Characterization of Multiple Calcium-Binding Sites and Calcium-Induced Conformational Changes. J. Biol. Chem..

[9] Welch R.A. (2000). RTX Toxin Structure and Function: A Story of Numerous Anomalies and Few Analogies in Toxin Biology. Curr. Top. Microbiol. Immunol..

[10] Guermonprez P., Khelef N., Blouin E., Rieu P., Ricciardi-Castagnoli P., Guiso N., Ladant D., Leclerc C. (2001). The Adenylate Cyclase Toxin of *Bordetella pertussis* Binds to Target Cells via the Alpha(M)Beta(2) Integrin (CD11b/CD18). J. Exp. Med..

[11] El-Azami-El-Idrissi M., Bauche C., Loucka J., Osicka R., Sebo P., Ladant D., Leclerc C. (2003). Interaction of *Bordetella pertussis* Adenylate Cyclase with CD11b/CD18. Role of Toxin Acylation and Identification of the Main Integrin Interaction Domain. J. Biol. Chem..

[12] Berkowitz S.A., Goldhammer A.R., Hewlett E.L., Wolff J. (1980). Activation of Prokaryotic Adenylate Cyclase by Calmodulin. Ann. N. Y. Acad. Sci..

[13] Confer D.L., Eaton J.W. (1982). Phagocyte Impotence Caused by an Invasive Bacterial Adenylate Cyclase. Science.

[14] Weingart C.L., Weiss A.A. (2000). *Bordetella pertussis* Virulence Factors Affect Phagocytosis by Human Neutrophils. Infect. Immun..

[15] Ehrmann I.E., Gray M.C., Gordon V.M., Gray L.S., Hewlett E.L. (1991). Hemolytic Activity of Adenylate Cyclase Toxin from *Bordetella pertussis*. FEBS Lett..

[16] Hewlett E.L., Donato G.M., Gray M.C. (2006). Macrophage Cytotoxicity Produced by Adenylate Cyclase Toxin from *Bordetella pertussis*: More than just Making Cyclic AMP!. Mol. Microbiol..

[17] Benz R., Maier E., Ladant D., Ullmann A., Sebo P. (1994). Adenylate Cyclase Toxin (CyaA) of *Bordetella pertussis* Evidence for the Formation of Small Ion-Permeable Channels and Comparison with HlyA of Escherichia Coli. J. Biol. Chem..

[18] Osicka R., Osickova A., Hasan S., Bumba L., Cerny J., Sebo P. (2015). Bordetella Adenylate Cyclase Toxin is a Unique Ligand of the Integrin Complement Receptor 3. eLife.

[19] Johnson M.S., Chouhan B.S. (2014). Evolution of Integrin I Domains. Adv. Exp. Med. Biol..

[20] Dal Molin F., Tonello F., Ladant D., Zornetta I., Zamparo I., Di Benedetto G., Zaccolo M., Montecucco C. (2006). Cell Entry and cAMP Imaging of Anthrax Edema Toxin. EMBO J..

[21] Carbonetti N.H. (2010). Pertussis Toxin and Adenylate Cyclase Toxin: Key Virulence Factors of *Bordetella pertussis* and Cell Biology Tools. Future Microbiol..

[22] Young J.A.T., Collier R.J. (2007). Anthrax Toxin: Receptor Binding, Internalization, Pore Formation, and Translocation. Annu. Rev. Biochem..

[23] Paccani S.R., Molin F.D., Benagiano M., Ladant D., D’Elios M.M., Montecucco C., Baldari C.T. (2008). Suppression of T-Lymphocyte Activation and Chemotaxis by the Adenylate Cyclase Toxin of *Bordetella pertussis*. Infect. Immun..

[24] Rogel A., Hanski E. (1992). Distinct Steps in the Penetration of Adenylate Cyclase Toxin of *Bordetella pertussis* into Sheep Erythrocytes. Translocation of the Toxin Across the Membrane. J. Biol. Chem..

[25] Basler M., Knapp O., Masin J., Fiser R., Maier E., Benz R., Sebo P., Osicka R. (2007). Segments Crucial for Membrane Translocation and Pore-Forming Activity of Bordetella Adenylate Cyclase Toxin. J. Biol. Chem..

[26] Linhartová I., Bumba L., Mašn J., Basler M., Osicka R., Kamanová J., Procházková K., Adkins I., HejnováHolubová J., Sadílková L. (2010). RTX Proteins: A Highly Diverse Family Secreted Bya Common Mechanism. FEMS Microbiol. Rev..

[27] Gordon V.M., Leppla S.H., Hewlett E.L. (1988). Inhibitors of Receptor-Mediated Endocytosis Block the Entry of Bacillus Anthracis Adenylate Cyclase Toxin but Not that of *Bordetella pertussis* Adenylate Cyclase Toxin. Infect. Immun..

[28] Osickova A., Masin J., Fayolle C., Krusek J., Basler M., Pospisilova E., Leclerc C., Osicka R., Sebo P. (2010). Adenylate Cyclase Toxin Translocates Across Target Cell Membrane without Forming a Pore. Mol. Microbiol..

[29] Osicková A., Osicka R., Maier E., Benz R., Šebo P. (1999). An Amphipathic a-Helix Including Glutamates 509 and 516 is Crucial for Membrane Translocation of Adenylate Cyclase Toxin and Modulates Formation and Cation Selectivity of its Membrane Channels. J. Biol. Chem..

[30] Masin J., Osicka R., Bumba L., Sebo P. (2015). Bordetella Adenylate Cyclase Toxin: A Unique Combination of a Pore-Forming Moiety with a Cell-Invading Adenylate Cyclase Enzyme. Pathog. Dis..

[31] Karst J.C., Enguéné V.Y.N., Cannella S.E., Subrini O., Hessel A., Debard S., Ladant D., Chenal A. (2014). Calcium, Acylation, and Molecular Confinement Favor Folding of *Bordetella pertussis* Adenylate Cyclase Cyaa Toxin. J. Biol. Chem..

[32] Cannella S.E., Ntsogo Enguéné V.Y., Davi M., Malosse C., Sotomayor Pérez A.C., Chamot-Rooke J., Vachette P., Durand D., Ladant D., Chenal A. (2017). Stability, Structural and Functional Properties of a Monomeric, Calcium-Loaded Adenylate Cyclase Toxin, CyaA, from *Bordetella pertussis*. Sci. Rep..

[33] Veneziano R., Rossi C., Chenal A., Devoisselle J.-M., Ladant D., Chopineau J. (2013). *Bordetella pertussis* Adenylate Cyclase Toxin Translocation Across a Tethered Lipid Bilayer. Proc. Natl. Acad. Sci. USA.

[34] Otero A.S., Yi X.B., Gray M.C., Szabo G., Hewlett E.L. (1995). Membrane Depolarization Prevents Cell Invasion by *Bordetella pertussis* Adenylate Cyclase Toxin. J. Biol. Chem..

[35] Gray M., Szabo G., Otero A.S., Gray L., Hewlett E. (1998). Distinct Mechanisms for K^+^ Efflux, Intoxication, and Hemolysis by *Bordetella pertussis* AC Toxin. J. Biol. Chem..

[36] Gmira S., Karimova G., Ladant D. (2001). Characterization of Recombinant *Bordetella pertussis* Adenylate Cyclase Toxins Carrying Passenger Proteins. Res. Microbiol..

[37] Holubova J., Kamanova J., Jelinek J., Tomala J., Masin J., Kosova M., Stanek O., Bumba L., Michalek J., Kovar M. (2012). Delivery of Large Heterologous Polypeptides Across the Cytoplasmic Membrane of Antigen-Presenting Cells by the Bordetella RTX Hemolysin Moiety Lacking the Adenylyl Cyclase Domain. Infect. Immun..

[38] Iwaki M., Konda T. (2016). Adenylate Cyclase Toxin-Mediated Delivery of the S1 Subunit of Pertussis Toxin into Mammalian Cells. Pathog. Dis..

[39] Fiser R., Masin J., Basler M., Krusek J., Spulakova V., Konopasek I., Sebo P. (2007). Third Activity of Bordetella Adenylate Cyclase (AC) Toxin-Hemolysin. Membrane Translocation of AC Domain Polypeptide Promotes Calcium Influx into CD11b^+^ Monocytes Independently of the Catalytic and Hemolytic Activities. J. Biol. Chem..

[40] Bumba L., Masin J., Fiser R., Sebo P. (2010). Bordetella Adenylate Cyclase Toxin Mobilizes its Beta2 Integrin Receptor into Lipid Rafts to Accomplish Translocation Across Target Cell Membrane in Two Steps. PLoS Pathog..

[41] Gordon V.M., Young W.W., Lechler S.M., Gray M.C., Leppla S.H., Hewlett E.L. (1989). Adenylate Cyclase Toxins from Bacillus Anthracis and *Bordetella pertussis*. Different Processes for Interaction with and Entry into Target Cells. J. Biol. Chem..

[42] Rogel A., Schultz J.E., Brownlie R.M., Coote J.G., Parton R., Hanski E. (1989). *Bordetella pertussis* Adenylate Cyclase: Purification and Characterization of the Toxic form of the Enzyme. EMBO J..

[43] Eby J.C., Gray M.C., Mangan A.R., Donato G.M., Hewlett E.L. (2012). Role of CD11b/CD18 in the Process of Intoxication by the Adenylate Cyclase Toxin of *Bordetella pertussis*. Infect. Immun..

[44] González-Bullón D., Uribe K.B., Martín C., Ostolaza H., Phospholipase A. (2017). Activity of Adenylate Cyclase Toxin Mediates Translocation of its Adenylate Cyclase Domain. Proc. Natl. Acad. Sci. USA.

[45] Guo Q., Shen Y., Lee Y.-S., Gibbs C.S., Mrksich M., Tang W.-J. (2005). Structural Basis for the Interaction of *Bordetella pertussis* Adenylyl Cyclase Toxin with Calmodulin. EMBO J..

[46] Karst J.C., Sotomayor Perez A.C., Guijarro J.I., Raynal B., Chenal A., Ladant D. (2010). Calmodulin-Induced Conformational and Hydrodynamic Changes in the Catalytic Domain of *Bordetella pertussis* Adenylate Cyclase Toxin. Biochemistry.

[47] Karst J.C., Barker R., Devi U., Swann M.J., Davi M., Roser S.J., Ladant D., Chenal A. (2012). Identification of a Region that Assists Membrane Insertion and Translocation of the Catalytic Domain of *Bordetella pertussis* CyaA Toxin. J. Biol. Chem..

[48] Gray M.C., Lee S.J., Gray L.S., Zaretzky F.R., Otero A.S., Szabo G., Hewlett E.L. (2001). Translocation-Specific Conformation of Adenylate Cyclase Toxin from *Bordetella pertussis* Inhibits Toxin-Mediated Hemolysis. J. Bacteriol..

[49] Subrini O., Sotomayor-Perez A.C., Hessel A., Spiaczka-Karst J., Selwa E., Sapay N., Veneziano R., Pansieri J., Chopineau J., Ladant D. (2013). Characterization of a Membrane-Active Peptide from the *Bordetella pertussis* CyaA Toxin. J. Biol. Chem..

[50] Yang J., Yan R., Roy A., Xu D., Poisson J., Zhang Y. (2014). The I-TASSER Suite: Protein Structure and Function Prediction. Nat. Methods.

[51] Masin J., Osickova A., Sukova A., Fiser R., Halada P., Bumba L., Linhartova I., Osicka R., Sebo P. (2016). Negatively Charged Residues of the Segment Linking the Enzyme and Cytolysin Moieties Restrict the Membrane-Permeabilizing Capacity of Adenylate Cyclase Toxin. Sci. Rep..

[52] Karimova G., Fayolle C., Gmira S., Ullmann A., Leclerc C., Ladant D. (1998). Charge-Dependent Translocation of *Bordetella pertussis* Adenylate Cyclase Toxin into Eukaryotic Cells: Implication for the in Vivo Delivery of CD8^+^ T Cell Epitopes into Antigen-Presenting Cells. Proc. Natl. Acad. Sci. USA.

[53] Rydel T.J., Williams J.M., Krieger E., Moshiri F., Stallings W.C., Brown S.M., Pershing J.C., Purcell J.P., Alibhai M.F. (2003). The Crystal Structure, Mutagenesis, and Activity Studies Reveal that Patatin is a Lipid Acyl Hydrolase with a Ser-Asp Catalytic Dyad. Biochemistry.

[54] Strickland J.A., Orr G.L., Walsh T.A. (1995). Inhibition of Diabrotica Larval Growth by Patatin, the Lipid Acyl Hydrolase from Potato Tubers. Plant Physiol..

[55] Grossman S., Oestreicher G., Singer T.P. (1974). Determination of the Activity of Phospholipases A, C, and D. Methods Biochem. Anal..

[56] Ros U., García-Sáez A.J. (2015). More than a Pore: The Interplay of Pore-Forming Proteins and Lipid Membranes. J. Membr. Biol..

[57] Gilbert R.J.C. (2016). Protein-lipid Interactions and Non-Lamellar Lipidic Structures in Membrane Pore Formation and Membrane Fusion. Biochim. Biophys. Acta (BBA) Biomembr..

[58] Basañez G., Sharpe J.C., Galanis J., Brandt T.B., Hardwick J.M., Zimmerberg J. (2002). Bax-Type Apoptotic Proteins Porate Pure Lipid Bilayers through a Mechanism Sensitive to Intrinsic Monolayer Curvature. J. Biol. Chem..

[59] Terrones O., Antonsson B., Yamaguchi H., Wang H.-G., Liu J., Lee R.M., Herrmann A., Basañez G. (2004). Lipidic Pore Formation by the Concerted Action of Proapoptotic BAX and tBID. J. Biol. Chem..

[60] Martin C., Requero M.A., Masin J., Konopasek I., Goni F.M., Sebo P., Ostolaza H. (2004). Membrane Restructuring by *Bordetella pertussis* Adenylate Cyclase Toxin, a Member of the RTX Toxin Family. J. Bacteriol..

[61] Bychkova V.E., Pain R.H., Ptitsyn O.B. (1988). The ‘Molten Globule’ State is Involved in the Translocation of Proteins Across Membranes?. FEBS Lett..

[62] Ptitsyn O.B., Pain R.H., Semisotnov G.V., Zerovnik E., Razgulyaev O.I. (1990). Evidence for a Molten Globule State as a General Intermediate in Protein Folding. FEBS Lett..

